# A sensitive ERK fluorescent probe reveals the significance of minimal EGF-induced transcription

**DOI:** 10.1247/csf.24070

**Published:** 2024-12-18

**Authors:** Zhang Weisheng, Jun Nakayama, Yukino Inomata, Shigeki Higashiyama, Toru Hiratsuka

**Affiliations:** 1 Department of Molecular Oncology, Graduate School of Medicine, Osaka University, Osaka, Japan; 2 Department of Oncogenesis and Growth Regulation, Research Center, Osaka International Cancer Institute, Osaka, Japan; 3 Division of Cell Growth and Tumor Regulation, Proteo-Science Center (PROS), Toon, Ehime, Japan; 4 Department of Biochemistry and Molecular Genetics, Ehime University Graduate School of Medicine, Toon, Ehime, Japan

**Keywords:** fluorescent probe, ERK, FRET, KTR

## Abstract

Extracellular signal-regulated kinase (ERK) regulates multiple cellular functions through distinct activation patterns. Genetically encoded fluorescent probes are instrumental in dissecting the ERK activity dynamics in living cells. Here we modified a previously reported Förster resonance energy transfer (FRET) probe for ERK, EKAREN5 by replacing its mTurquoise2 and YPet sequences with mTurquoise-GL and a synonymous codon variant of YPet, respectively. The modified biosensor, EKAREN5-gl, showed an increased sensitivity to EGF-induced ERK activation responding to a very low dose (20 pg/ml) of EGF stimulation. We quantitatively characterized two FRET-based ERK probes, EKAREN5 and EKAREN5-gl, and a subcellular kinase translocation-based probe, ERK-KTR. We found the three biosensors differently respond to EGF stimulations with different intensity, duration, and latency. Furthermore, we investigated how the minimal EGF-induced ERK activation affects the downstream transcription in HeLa cells by comprehensive transcriptional analysis. We found the minimal ERK activation leads to a distinct transcriptional pattern from those induced by higher ERK activations. Our study highlights the significance of sensitive fluorescent probes to understand cellular signal dynamics and the role of minimal ERK activation in regulating transcription.

## Graphical Abstract

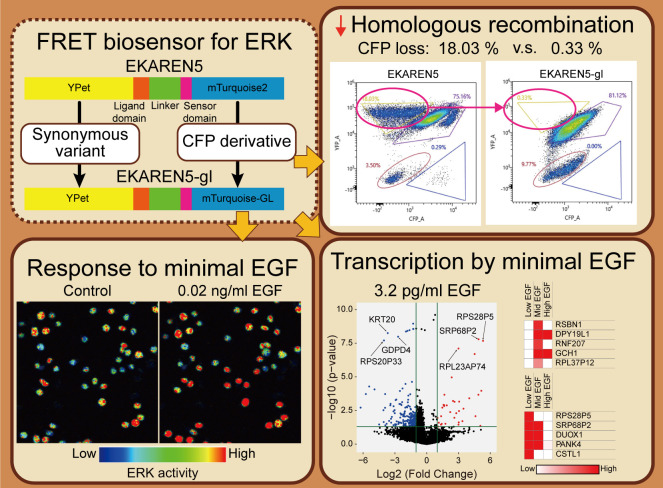

## Introduction

ERK (extracellular signal-regulated kinase) is a classical serine/threonine kinase that regulates multiple cellular functions including cell proliferation, migration, differentiation, and metabolism (Fang and Richardson, 2005; Lavoie *et al.*, 2020; Ramos, 2008). ERK signaling pathway is initiated by extracellular growth factors such as EGF (epidermal growth factor) and mediated by a small GTPase RAS. RAS recruits RAF to the plasma membrane, which triggers phosphorylation relay of RAF and MEK, which eventually culminates in the activation of ERK and downstream transcriptional programs. This intricate signaling network plays a key role in physiological tissue maintenance and deregulation of ERK signaling pathway leads to numerous pathological conditions such as cancer (Dhillon *et al.*, 2007; Guo *et al.*, 2020; Kim and Choi, 2010; Roberts and Der, 2007). Therefore, ERK has been a central molecular target in understanding the tissue homeostasis and the development of therapeutics of diseases.

Fluorescent probes are powerful tools to link the intricate ERK activity dynamics to cellular behaviors at single-cell resolution. Since the first development of Förster resonance energy transfer (FRET)-based probe for ERK, EKAR (extracellular signal-regulated kinase activity reporter) (Harvey *et al.*, 2008), modifications of EKAR have enabled monitor ERK activity with higher sensitivity and specificity, which includes EKAREV (extracellular signal-regulated kinase activity reporter-Eevee-linker) (Komatsu *et al.*, 2011), EKAR-rEV (Lin *et al.*, 2022), and EKAREN5 (Ponsioen *et al.*, 2021). Additionally, recent development of KTR (kinase translocation reporter)-based ERK probes (Regot *et al.*, 2014; Tsai *et al.*, 2024; Wilcockson *et al.*, 2023) paved a new path in visualizing ERK activity at single cell resolution. These advancements of genetically encoded fluorescence probes led to the observation of various spatiotemporal ERK activity dynamics such as pulse activations (Albeck *et al.*, 2013; Aoki *et al.*, 2013) and propagations (Aoki *et al.*, 2017; Hiratsuka *et al.*, 2015; Ram *et al.*, 2023a, 2023b). These findings suggest even transient and slight activation of ERK can trigger transcriptional programs to regulate cell proliferation, migration, and differentiation.

Here, we report EKAREN5-gl, which is based on a previously-reported ERK probe, EKAREN5. EKAREN5-gl has a reduced genetic homology between the two fluorescent protein sequences. We found EKAREN5-gl allows us to detect slight ERK activations induced by low-dose EGF stimulation that EKAREN5 and ERK-KTR could not detect. Furthermore, we examined the transcriptional effect of minimal ERK activation by comprehensive transcriptional analysis, revealing a distinctive transcription in low EGF stimulation from that in higher EGF stimulations. Our study not only provides a new tool for detecting minimal ERK activities but also sheds light on their transcriptional significance.

## Material and Methods

### Reagent

EGF was purchased from Thermo Fisher Scientific, Waltham, MA, USA. PD0325901 was purchased from Funakoshi Co., Ltd., Tokyo, Japan. SiR-DNA was purchased from Cytoskeleton, Inc., Denver, CO, USA.

### Plasmids

Lentiviral plasmids for EKAREN5 and ERK-KTR-mClover were obtained from Addgene (#167822 and #59150). EKAREN5-gl lentiviral plasmid was constructed by replacing the YPet and mTurquoise2 sequence cassettes with a synonymous codon variant of YPet and mTurquoise-GL (Goedhart *et al.*, 2010), respectively by In-Fusion HD Cloning Kit (Takara Bio, Inc., Kusatsu, Japan). Plasmids where the CFP or YFP sequence was removed from EKAREN5 were constructed by In-Fusion HD Cloning Kit (Takara Bio, Inc.). The synonymous codon variant of YPet cassette was obtained from EKARrEV-NLS by PCR (Lin *et al.*, 2022).

### Cell culture

HeLa cells were purchased from the Human Science Research Resources Bank, Japan and cultured in DMEM (Nacalai Tesque, Inc., Kyoto, Japan.) supplemented with 10% fetal bovine serum (Nichirei Biosciences, Inc., Tokyo, Japan) under 37°C and 5% CO_2_. For live imaging, HeLa cells were plated on an 8-well chamber slide (Iwaki AGC Techno Glass CO., LTD, Haibara-gun, Japan) coated with Cellmatrix Type-I-C solution (Nitta Gelatin, Inc., Yao, Japan) at 37°C for 1 hour.

### Lentivirus production and infection

Lenti-X 293T cells (Takara Bio, Inc.) were cultured in DMEM (Nacalai Tesque, Inc.) supplemented with 10% FBS (Nichirei Biosciences Inc.). Lenti-X 293T cells with 70–90% confluency were co-transfected with pCSII lentiviral plasmid, pCMV-VSV-G-RSV-Rev (RIKEN, Tsukuba, Japan) and psPAX2 (Addgene #12260). After 2 days of incubation at 37°C with 5% CO_2_, viruses were collected and concentrated by Lenti-X Concentrator (Takara Bio, Inc.). For lentivirus infection, HeLa cells were transduced with the concentrated virus solution under 37°C with 5% CO_2_ for 1 day.

### Stable cell lines

To establish HeLa cell lines stably expressing genetically-encoded fluorescence probes, HeLa cells infected with lentivirus encoding the probes were cloned by limiting dilution cloning.

### DNA transfection

For transfection, cells were transfected with DNA plasmids using Polyethyleneimine (Selleck Chemicals LLC., Houston, TX, USA).

### Cell spectrum analysis

Fluorescence spectrum of HeLa cells expressing fluorescence probe was analyzed by spectrum cell analyzer SA3800 (Sony Biotechnology, Inc., Tokyo, Japan), which is equipped with 405 nm, 488 nm, 561 nm, and 638 nm excitation lasers and Violet x2ch PMT (420–440 nm/450–469 nm) and 32chPMT (500–800 nm) detectors. Cells were suspended in PBS supplanted with 3% fetal bovine serum (Nichirei Biosciences, Inc.) and 1% penicillin streptomycin (Nacalai Tesque, Inc.). The expressions of CFP and YFP were analyzed by spectrum compensation in SA3800 software (Sony Biotechnology, Inc.) using HeLa cells expressing only CFP or YFP. The software allows for CFP and YFP signal analysis by its spectrum unmixing algorithm of whole fluorescence spectrum covering 420–440 nm, 450–469 nm, and 500–800 nm range. 50,000 cells derived from a single cell culture plate were analyzed for each cell line.

### Microscope

Images were acquired with FLUOVIEW FV10i confocal microscope (Olympus, Tokyo, Japan). CFP and YFP-based FRET biosensors were imaged by preset dye settings, ECFP and EYFP, in Fluoview software (Olympus). ERK-KTR and nuclear SiR-DNA label were imaged by preset dye settings, EGFP and FarRed.

### Image acquisition

HeLa cells expressing fluorescence probes were starved for serum for 1 hour in FluoroBrite^TM^ DMEM (Life Technologies Corporation., Carlsbad, CA, USA) supplemented with 2 mmol/L L-Alanyl-L-glutamine Solution (Nacalai Tesque, Inc.) at 37°C and 5% CO_2_. For nuclear labeling in cells expressing ERK KTR, the cells were pre-treated with SiR-DNA dye for 40 minutes before serum starvation. Images were acquired every 2 minutes.

### Image analysis

Images were processed and analyzed with Fiji/ImageJ (https://imagej.net/Fiji). Cells expressing FRET probes were segmented by ImageJ plugin, TrackMate (Ershov *et al.*, 2022). Cells expressing ERK-KTR biosensor and stained with SiR-DNA were manually segmented for nuclear and cytoplasmic regions by Fiji/ImageJ. Ratiometric FRET/CFP values in FRET biosensors and cytoplasm-to-nucleus ratio in ERK-KTR probe were analyzed using Excel software (Microsoft, Redmond, WA, USA).

### RNA sequencing

Total RNAs were extracted and purified using the RNeasy Mini Kit (Qiagen NV, Venlo, Netherlands) from HeLa cells treated with 0.0032 ng/ml EGF, 0.08 ng/ml EGF, 10 ng/ml EGF or DDW for 5 hours. The quality of RNA was assessed using 2100 Bioanalyzer (Agilent Technologies, Tokyo, Japan). RNA concentration was measured using a Qubit 2.0 Fluorometer (Life Technologies Corporation). Illumina sequencing libraries were constructed using the NEBNext Ultra II Directional RNA Library Prep Kit for Illumina (New England Biolabs, Ipswich, MA, USA) and sequenced on DNBSEQ-T7 by pair-end sequencing with a read length of 2 × 150 bp by Novogene, Beijing, China. Expression levels for each gene were quantified from the sequencing data using Kallisto (Bray *et al.*, 2016). The data were then summarized using the tximport package (ver. 1.18.0) of R software (ver. 4.0.3) and RStudio (RStudio), and scaledTPM counts were used for further analysis as expression values. Principal component analysis (PCA) was performed by R software (ver. 4.4.1).

### Statistical analysis

Excel software (Microsoft) was used for all the statistical analyses. Student’s t-test was used to evaluate significant differences. For duplicated t-test analysis, the values were subjected to One-way ANOVA test and the p-values were corrected by Bonferroni correction. P-values less than 0.05 were considered statistically significant (*P<0.05, **P<0.01, ***P<0.001).

### Data availability

The data in this study are available from the corresponding author upon reasonable request.

## Results

### EKAREN5-gl has a reduced recombination between the CFP and YFP sequences

**Fig. 1:**
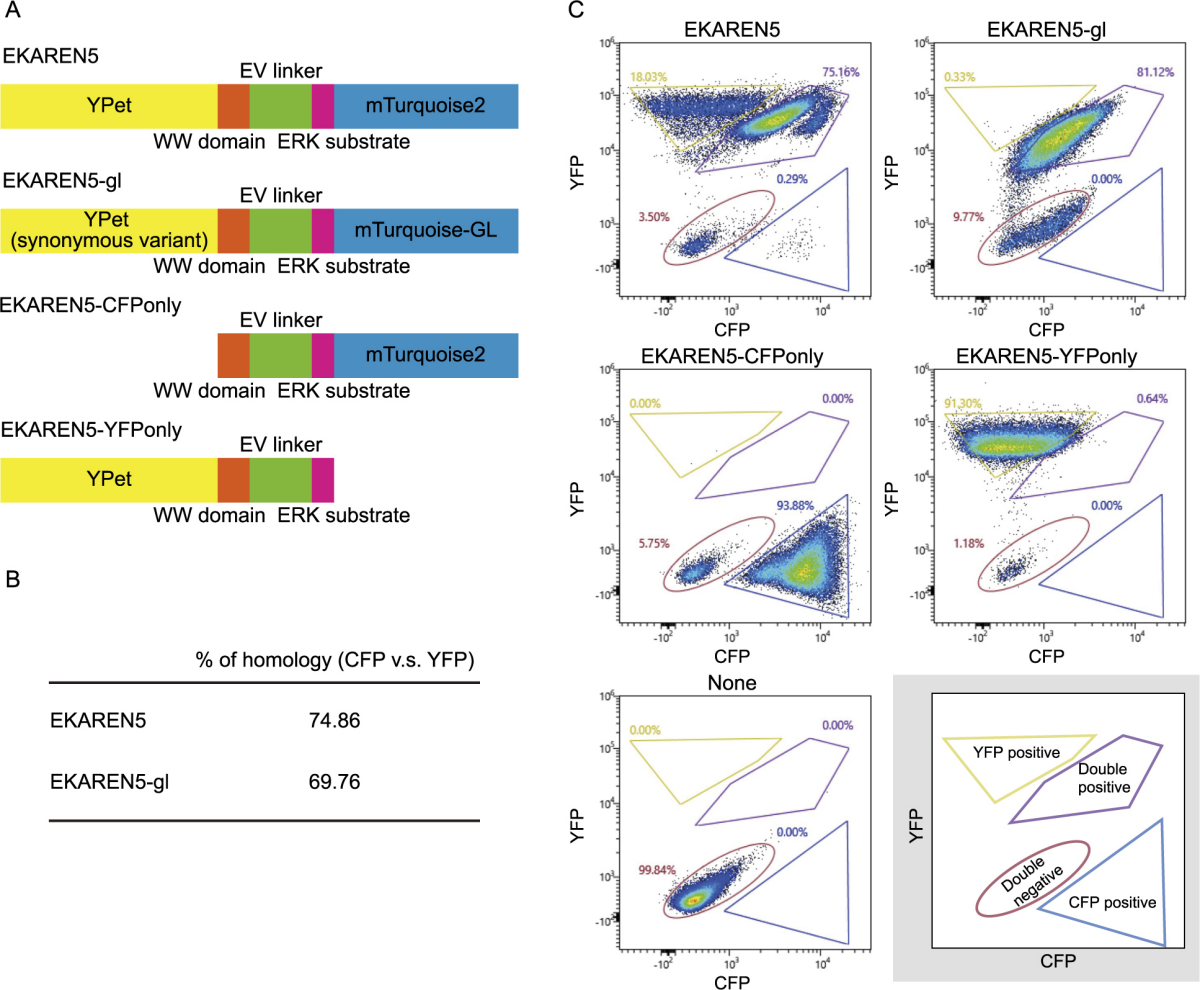
Reduced recombination between CFP and YFP sequences in EKAREN5-gl. (A) Schematics of the original EKAREN5, EKAREN5-gl, a modified EKAREN5 probe where its mTurquoise2 and YPet sequences were replaced with mTurquoise-GL and a synonymous codon variant YPet, EKAREN5 without YPet sequence (EKAREN5-CFPonly), and EKAREN5 without mTurquoise2 (EKAREN5-YFPonly). Note that the schematics of EKAREN5 and EKAREN5-gl were previously used (Tsukamoto *et al.*, in press). (B) Homology rate between CFP and YFP gene sequences in EKAREN5 and EKAREN5-gl. (C) Single cell expression profile of CFP and YFP in HeLa cells expressing the indicated fluorescent probes analyzed by cell spectrum analyzer. Gates for cells expressing only CFP or YFP were determined by the profile of EKAREN5-CFPonly and EKAREN5-YFPonly. The right bottom panel (gray background) schematically shows each cell population.

Homologous recombination in single-molecule FRET probes is a drawback for stable expression of the probe by lentiviral integration into host cell genome (Aoki *et al.*, 2012; Komatsubara *et al.*, 2015; Terai *et al.*, 2019). High homology between the two fluorescent protein sequences is the major cause of genetic recombination and it leads to loss of expression of either of the two fluorescent proteins. Aiming to reduce the sequence homology of EKAREN5 ERK probe, we created EKAREN5-gl, where the YPet and mTurquoise2 sequences in the original EKAREN5 probe were replaced with a synonymous codon variant YPet (Fig. S1) and mTurquoise-GL. We sought to examine the intra-probe recombination rate by cell fluorescence spectrum analysis. We constructed fluorescence probes expressing only CFP or YFP to mimic the loss of fluorescence caused by homologous recombination (Fig. 1A). We found EKAREN5-gl has a reduced CFP-YFP sequence homology of 69.76% compared to that of 74.86% in EKAREN5 (Fig. 1B). HeLa cells transduced with lentivirus encoding the fluorescent probes were subjected to fluorescence spectrum analysis to examine the rate of homologous recombination (Fig. 1C). We found 18.03% of HeLa cells expressing EKAREN5 show only YFP fluorescence but not CFP, which suggests loss of CFP fluorescence by homologous recombination. In contrast, HeLa cells expressing EKAREN5-gl did not show loss of CFP fluorescence (0.33%). This indicates that EKAREN5-gl is beneficial for lentiviral stable expression in the host cells.

### The response of EKAREN5-gl to EGF-induced ERK activation is comparable to EKAREN5

**Fig. 2:**
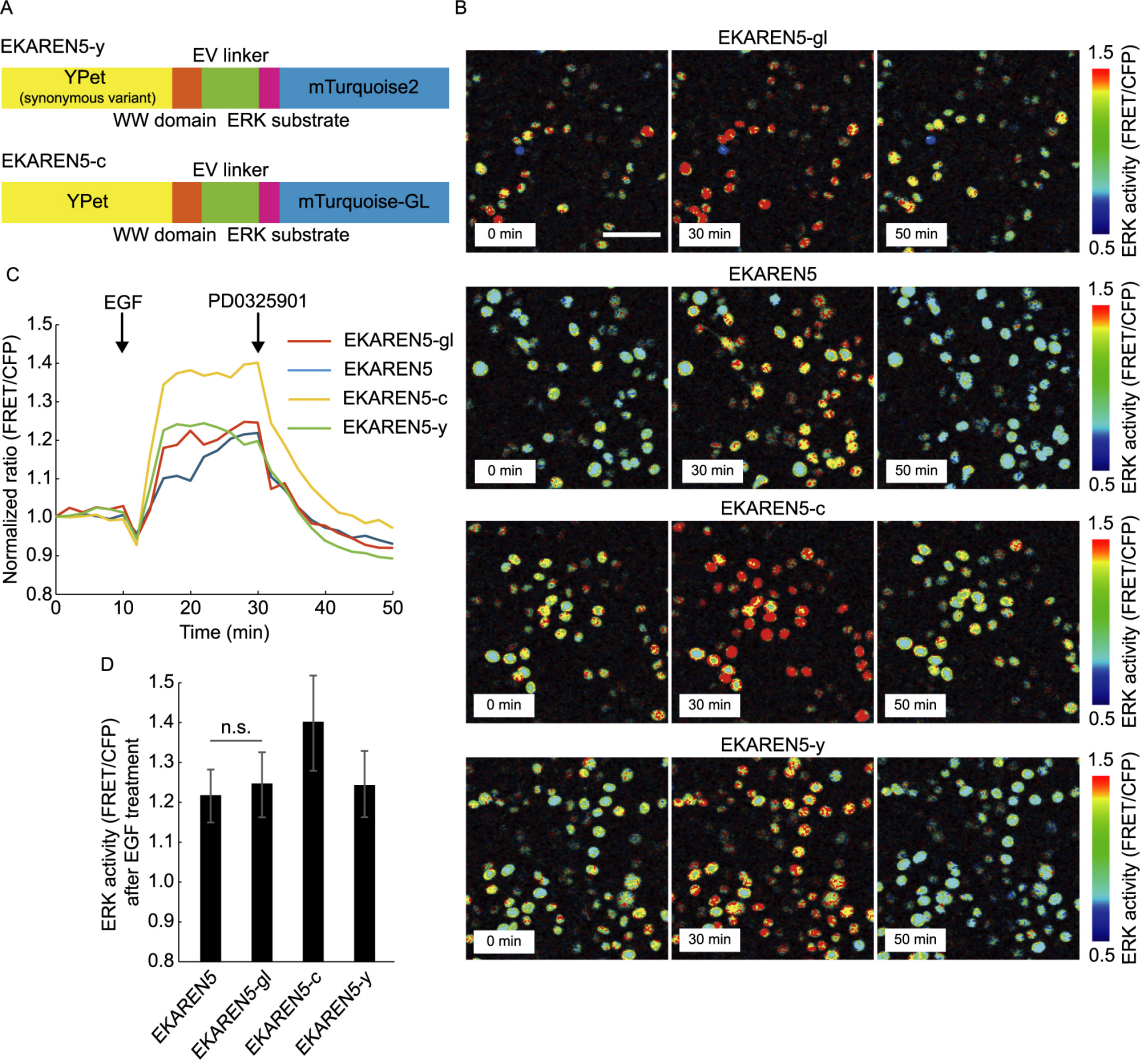
The response of EKAREN5-gl is comparable to EKAREN5. (A) Schematics of EKAREN5-y and EKAREN5-c, where only YPet or mTurquoise2 in EKAREN5 was replaced with a synonymous codon variant YPet or mTurquoise-GL. (B) Intensity modulated display (IMD) images of ratiometric FRET ratio (FRET/CFP) in HeLa cells expressing EKAREN5-c, EKAREN5-y, EKAREN5 or EKAREN5-gl. Cells were treated with 10 ng/ml EGF at 10 min and 10 μM PD0325901 at 30 min after the initial image acquisition. Scale, 100 μm. (C) Time series of average ratiometric FRET ratio (FRET/CFP) in the HeLa cells expressing the indicated fluorescent probe (*n* = 181–277 cells). The values are normalized by the average FRET ratio before EGF addition. (D) The peak normalized FRET ratios (FRET/CFP) in HeLa cells shown in (C) expressing the indicated fluorescent probe after EGF treatment. Data are shown by mean ± SD. n.s.: not significant.

To examine the functionality of EKAREN5-gl as an ERK activity probe, we pharmacologically tested its responses to EGF-induced ERK activation and MEK inhibitor induced ERK inactivation. To test the effect of replacing either CFP or YFP sequence, we constructed probes where only mTurquioise2 or YPet sequence in EKAREN5 was replaced with mTurquoise-GL (EKAREN5-c) or a synonymous codon variant YPet (EKAREN5-y) (Fig. 2A). Upon 10 ng/ml EGF treatment, HeLa cells expressing EKAREN5-gl increased ratiometric FRET efficiency (FRET/CFP) by 24.6%, which is comparable to the original FRET probe, EKAREN5 (21.8%) (Fig. 2B, C). We found both EKAREN5-c and EKAREN5-y also shows significant increase in FRET/CFP ratio by EGF treatment. Furthermore, all the four FRET probes showed immediate decrease in the ratiometric FRET efficiency by the treatment of MEK inhibitor, PD0325901, confirming the specificity to the FRET biosensors to ERK activity (Fig. 2B–D). This confirms that the functionality of the EKAREN5 is not compromised by replacing the fluorescent proteins.

### EKAREN5-gl shows improved sensitivity to minimal EGF-induced ERK activation

**Fig. 3:**
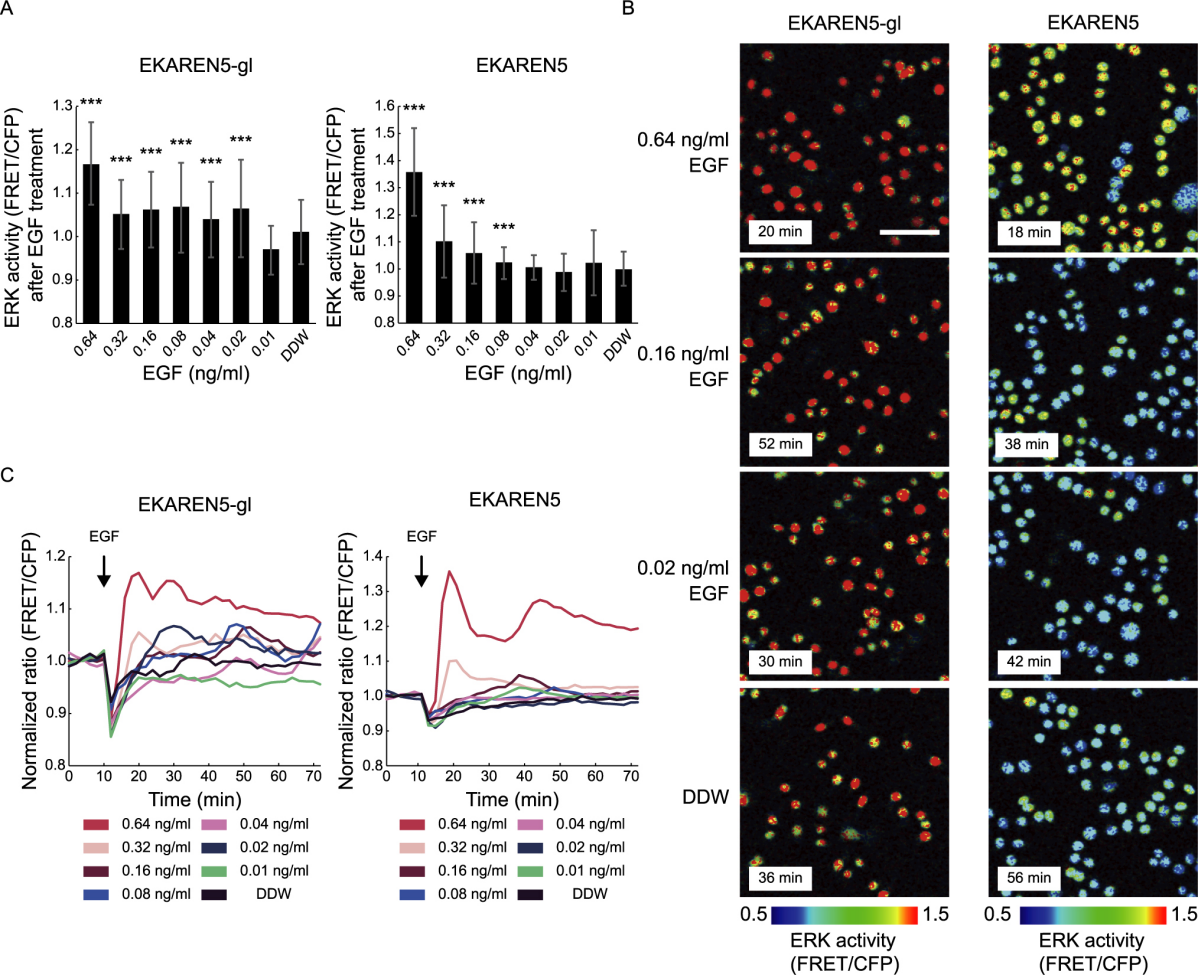
EKAREN5-gl allows detecting minimal EGF-induced ERK activation. (A) The peak FRET ratios (FRET/CFP) in HeLa cells expressing EKAREN5-gl (left, *n* = 135–239 cells) and EKAREN5 (right, *n* = 232–300 cells) after EGF treatment. The values are normalized by the average FRET ratio before EGF addition. Data are shown by mean ± SD. (B) IMD images of ratiometric FRET ratios (FRET/CFP) in HeLa cells expressing EKAREN5-gl (left) or EKAREN5 (right). Cells were treated with the indicated dose of EGF at 10 min. Scale, 100 μm. Images shown are from the timepoint of the peak FRET/CFP ratio. (C) Time series of average ratiometric FRET ratio (FRET/CFP) in HeLa cells expressing the EKAREN5-gl (left) or EKAREN5 (right) shown in A stimulated with the indicated dose of EGF. The values are normalized by the average FRET ratio before EGF addition.

**Fig. 4:**
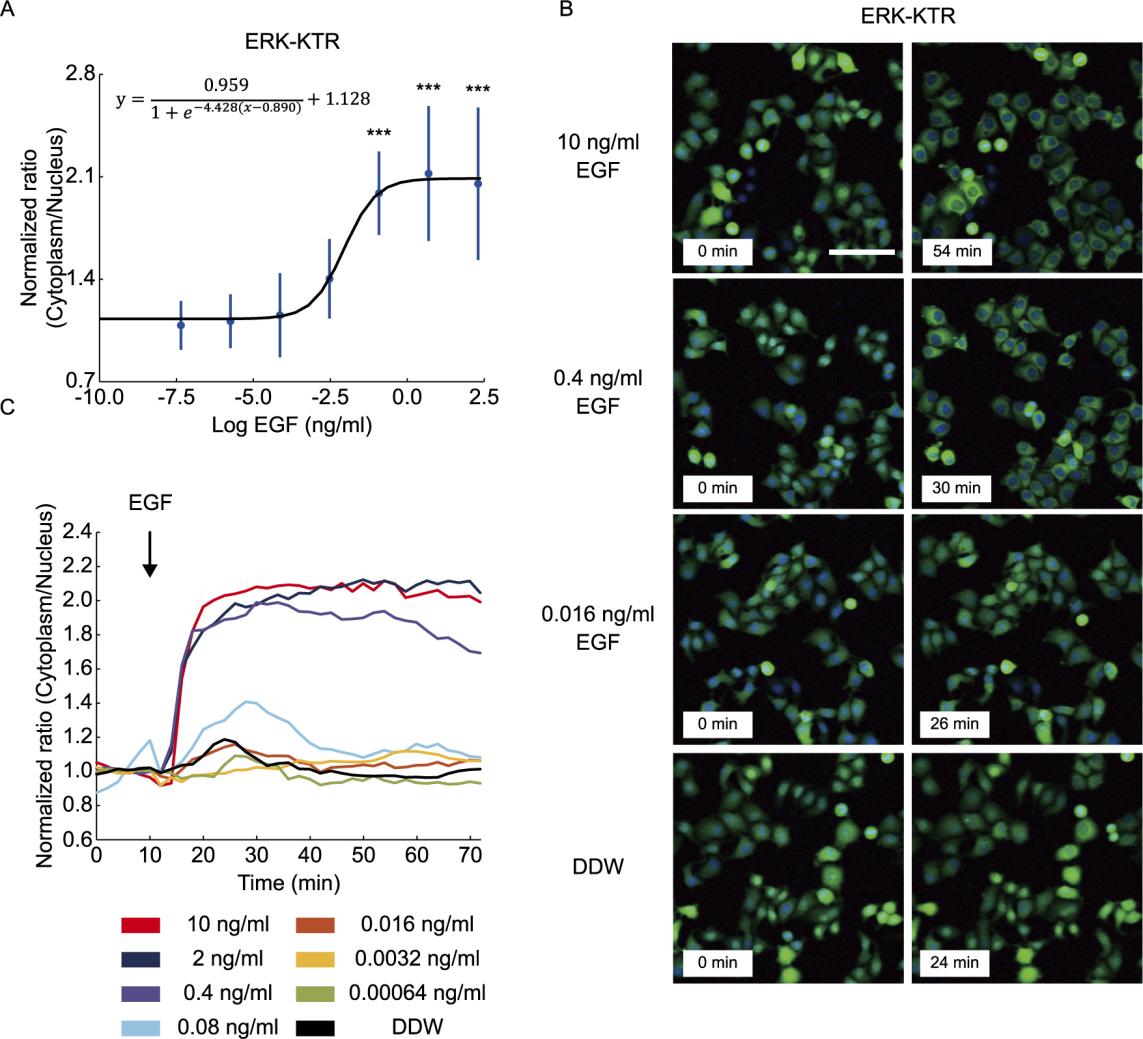
ERK-KTR probe is sensitive to ERK activation induced by relatively high doses of EGF stimulation. (A) The peak Cytoplasm/Nucleus ratios in HeLa cells expressing ERK-KTR after EGF treatment (*n* = 20 cells for each condition). The values are normalized by the average FRET ratio before EGF addition. Data are shown by mean ± SD. Black line shows a sigmoid curve fitting (fitting function is shown above). (B) Images of HeLa cells expressing ERK-KTR before and after EGF treatment. Cells were treated with the indicated dose of EGF at 10 min. Scale, 100 μm. Images shown are from the timepoint of the peak FRET/CFP ratio. (C) Time series of average cytoplasm/nucleus ratio in HeLa cells expressing ERK-KTR and stimulated with the indicated dose of EGF (*n* = 20 cells for each condition). The values are normalized by the average FRET ratio before EGF addition.

The high sensitivity of the FRET probes based on EKAREN5 propelled us to examine the minimum EGF stimulation detectable by the probes. We initially tested 0.00064–10 ng/ml EGF stimulation in HeLa cells expressing EKAREN5-gl. We found EKAREN5-gl shows a dose-dependent response to EGF. Among the EGF stimulations, 0.08 ng/ml or higher EGF stimulation showed a statistically significant increase in the FRET/CFP FRET ratio (Fig. S2). To further narrow down the minimum detectable EGF concentration with the probe, we tested 0.01–0.64 ng/ml EGF stimulation in HeLa cells expressing EKAREN5 or EKAREN5-gl. We found both EKAREN5-gl and EKAREN5 can detect ERK activations at low EGF concentrations in a dose-dependent manner (Fig. 3A). Consistently, the proportion of cells that responded to the EGF stimulation decreased by reduced EGF concentration (Fig. 3B). Our time-series analysis revealed that while high EGF stimulation causes immediate ERK activity response, low EGF stimulation led to delayed peak activity of FRET/CFP ratio (Fig. 3C). The minimum EGF-induced ERK activation detectable by EKAREN5-gl was that induced by 0.02 ng/ml EGF. This is significantly lower than the minimum detectable EGF dose of 0.08 ng/ml in EKAREN5. This indicates EKAREN5-gl is more sensitive than EKAREN5 in detecting slight ERK activity changes. For comparison, we tested another genetically-encoded ERK biosensor, ERK-KTR, which is based on kinase translocation (Regot *et al.*, 2014). As expected, ERK-KTR showed significant increase in cytoplasm-to-nucleus (C/N) ratio at the EGF concentration of 0.4 ng/ml or higher (Fig. 4A–C). This suggests that ERK-KTR has a similar sensitivity to ERK activity to the FRET biosensors. However, the gain of C/N ratio sharply dropped when the EGF concentration was lower than 0.4 ng/ml (Fig. 4A), which suggests a possible advantage of FRET biosensors to linearly detect gradual ERK activations. On the other hand, ERK-KTR was highly beneficial in detecting intense ERK activations by the visually evident translocation of fluorescence from nucleus to cytoplasm (Fig. 4B). Although the three genetically-encoded ERK probes can detect ERK activity upon a wide-range of EGF stimulations, the temporal patterns of the probes were different with ERK-KTR (Fig. 4C), showing relatively slow responses in both the activation and inactivation phase of the ERK activity, suggesting their differences in stoichiometric interactions with endogenous ERK and phosphatase members. Together, we show EKAREN5-gl allows detecting slight ERK activations induced by minimal EGF stimulation and the three different ERK probes respond to the ERK activation by different gain, latency, and reversibility.

### Minimal EGF stimulation induces distinct transcriptional signature

**Fig. 5:**
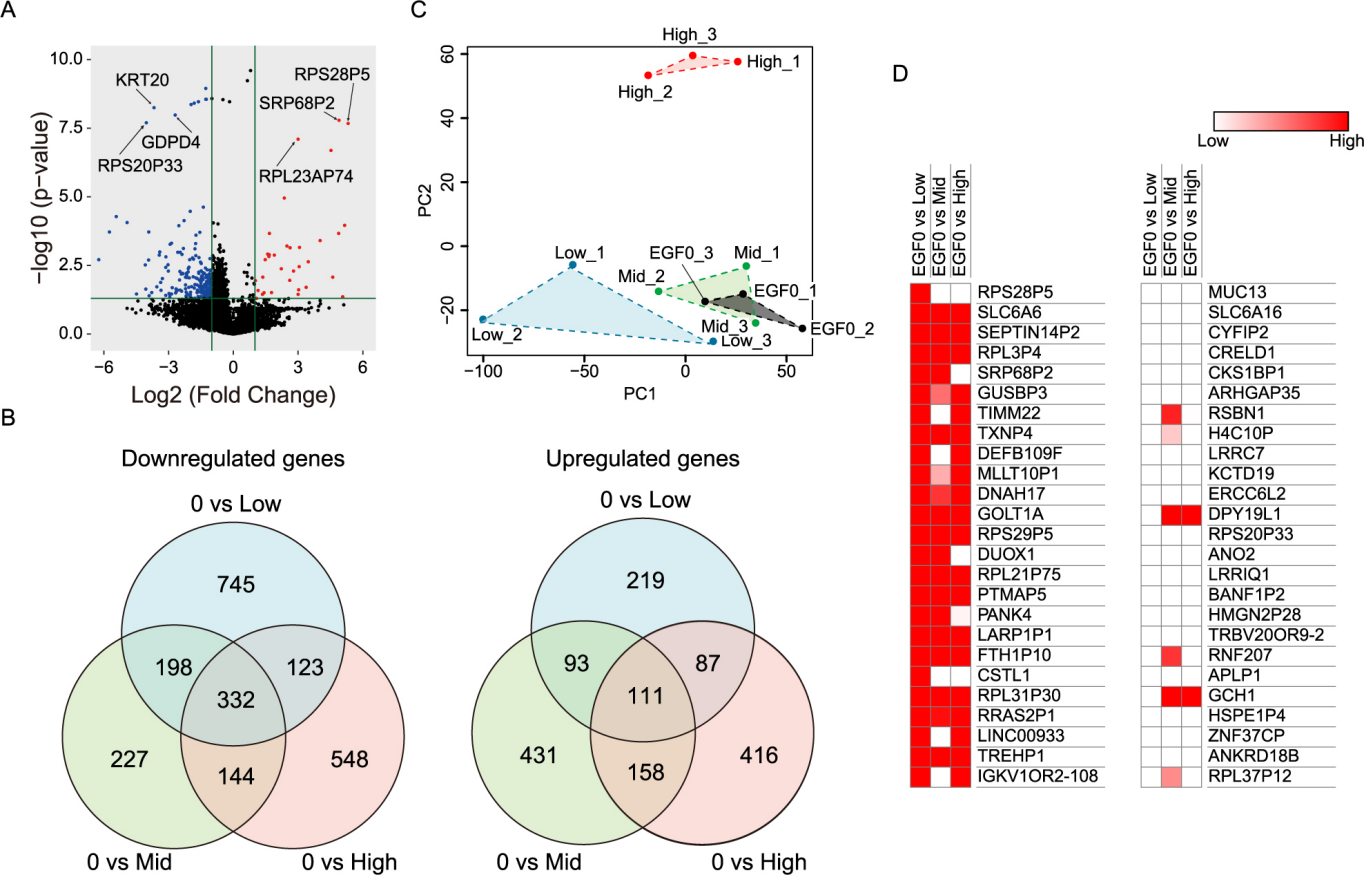
Minimal EGF stimulation induces a distinct transcriptional signature from high EGF stimulation. (A) Volcano plot showing differentially expressed genes in HeLa cells treated with low-dose (0.0032 ng/ml) EGF. (B) Venn diagram showing the number of commonly or distinctly upregulated (right) or downregulated (down) genes in the three comparison pairs (0: no EGF stimulation, Low: 0.0032 ng/ml EGF, Mid: 0.08 ng/ml EGF, High: 10 ng/ml EGF). (C) Principal component analysis (PCA) of differentially expressed genes in HeLa cells stimulated with low (0.0032 ng/ml), middle (0.08 ng/ml), or high (10 ng/ml)-dose EGF or DDW. Triplicates were analyzed for each condition. (D) Top 25 upregulated (left) and downregulated (right) genes in HeLa cells treated with low-dose EGF (0.0032 ng/ml) and their expressions in the middle (0.08 ng/ml) or high (10 ng/ml)-dose EGF treatment.

Given the high sensitivity of EKAREN5-gl to minimal EGF-induced ERK activation, we investigated the transcriptional effects of ERK activation induced by three different EGF doses: high and reliably detectable (10 ng/ml), intermediate and barely detectable (0.08 ng/ml), and low and not detectable (0.0032 ng/ml). We found HeLa cells with the lowest EGF dose still show significant induction of gene expressions including those related to ribosomal functions and protein synthesis (RPS28P5, SRP68P2, and RPL23AP74) (Fig. 5A). While some genes were commonly upregulated or downregulated in cells treated with different EGF doses, we found 745 genes and 219 genes were distinctively upregulated or downregulated in cells treated with the low EGF dose (0.0032 ng/ml) (Fig. 5B). This suggests that ERK activations of different degrees have distinct transcriptional effects to induce different cellular outcomes. Consistently, our principal component analysis (PCA) revealed the transcriptional profile induced by low EGF (0.0032 ng/ml) is distantly located from the axis from no EGF stimulation to high EGF-induced transcription (10 ng/ml). Interestingly, the triplicates of low-EGF stimulation showed relatively varied signatures compared to higher EGF stimulation (Fig. 5C). This may suggest the ERK-induced transcription is temporally fluctuating, which corroborates the significance of fluctuating ERK activity to regulate multiple cellular outcomes (Albeck *et al.*, 2013; Aoki *et al.*, 2013; Hiratsuka *et al.*, 2015, 2020; Miao and Pourquié, 2024). Among the distinctively upregulated genes in low EGF treatment, we found genes related to anti-tumor immunity, sperm flagellar assembly, and mitogenesis (SLC6A6, DNAH17, DUOX1) (Ashtiwi *et al.*, 2021; Shentu *et al.*, 2024; Song *et al.*, 2023). Conversely, distinctively downregulated genes included MUC13, CYFIP2, and ARHGAP35, which regulates cell signaling, apoptosis, and cell growth (Biembengut *et al.*, 2021; Héraud *et al.*, 2019; Reynolds *et al.*, 2019) (Fig. 5D). In line with our PCA analysis, the genes were not necessarily upregulated or downregulated in cells treated with higher EGF doses. Together, our transcriptional analyses show the biological significance of low ERK activation that may not be detectable with current state-of-the-art fluorescent probes. This suggests the need for even more sensitive methods to detect slight ERK activations that potentially elucidate the intricate cellular signaling network.

## Discussion

The reliability of genetically-encoded fluorescent probes is key for single-cell interrogation of signaling pathway. Besides the expression level and signal-to-noise ratio of the fluorescent probes, FRET-based probes may suffer from low sensitivity, low specificity, and unexpected recombination within the probe sequence. Here, we report EKAREN5-gl, which shows a high sensitivity to minimal ERK activity. Because we used a sub-optimized microscope set-up including laser wavelength (405 nm rather than 440 nm) and filter settings (pre-set CFP and YFP detection filters rather than customized wavelength range), the ERK probe may show even more sensitivity in a more sophisticated imaging set-up. Our validation of FRET probes in a sub-optimized set-up supports the applicability of the ERK probes to a wide range of researchers, not limited to specialists in microscopy and live imaging. Furthermore, the reduced sequential homology between the two fluorescent proteins in EKAREN5-gl significantly reduced the risk of homologous recombination that often disturbs ERK activity monitoring in lentivirally transduced cells (Aoki *et al.*, 2012; Komatsubara *et al.*, 2015; Terai *et al.*, 2019). Together, EKAREN5-gl and the future development of even more sensitive ERK probes will be highly valuable in understanding the intricate link between signal networks and single-cell behaviors.

Despite the continuous development and improvement of genetically-encoded ERK probes, users need to note that the probes are indirectly monitoring ERK activity by probe interactions with endogenous ERK and phosphatases. This leaves a concern that the endogenous ERK activity may differ from the ERK activity monitored by ERK probes. Indeed, the ERK activity monitored by the three probes, EKAREN5, EKAREN5-gl, and ERK-KTR differed not only in their gain of ratio (FRET/CFP or Cytoplasm/Nucleus signal) but also in latency, activation and inactivation period, and fluctuations. This suggests that reliable measurements of ERK activity require comparisons of multiple fluorescent probes and other methods including kinase-dead negative control FRET probes, FLIM (fluorescence lifetime imaging microscopy), immunoblotting, and immunohistochemistry.

Recent studies show temporal ERK activity patterns have significant biological roles in regulating cell proliferation, migration, differentiation, and tissue homeostasis (Hommes, 2003; Huang *et al.*, 2004; Khavari and Rinn, 2007; Muta *et al.*, 2019). This supports even slight ERK activity changes monitored in ERK probes are biologically significant. Indeed, we found even the non-detectable EGF stimulation (3.2 pg/ml) leads to significant transcriptional changes, which is not necessarily a downsized expression pattern of higher EGF concentrations.

Together, our development of EKAREN5-gl extends applications of genetically-encoded fluorescent probes for ERK and highlights the biological significance of ERK activities that are barely detectable with the current probes. While continuous efforts have been made to develop reliable ERK probes, image analysis has been another drawback for the application of the probes to a wide range of researchers (Ponsioen *et al.*, 2021). Nevertheless, future advancement both in image analyses such as artificial intelligence (Yousif *et al.*, 2022), machine learning (Kan, 2017; Seo *et al.*, 2020) and ERK probes such as ERK-nKTR, which monitors ERK activity solely by its nuclear signal will be promising in sensitively detecting ERK activity and finding its biological relevance.

## Funding

This work was financially supported in part by grants awarded to Toru Hiratsuka.

• Institution awarded the funding: Osaka International Cancer Institute

• Funder: JSPS KAKENHI Grant-in-Aid for Scientific Research (C), Japan; JSPS KEKENHI Grant-in-Aid for Early Career Scientists, Japan; Konica Minolta Imaging Science Encouragement Award, Japan; The Mitsubishi Foundation, Japan

• Grant number: 24K10301 (JSPS KAKENHI Grant-in-Aid for Scientific Research (C)); 22K15510 (JSPS KEKENHI Grant-in-Aid for Early Career Scientists)

## Conflict of Interest

The authors have no conflict of interest to declare.

## Ethics Statement

We have no ethics statement to declare.

## Author Contributions

T. Hiratsuka conceived the idea. Z. Weisheng, Y. Inomata, and T. Hiratsuka performed the experiments including cell culture, DNA construction, and live imaging. J. Nakayama analyzed RNA sequence data. S. Higashiyama helped supervising the project. All authors discussed the results and contributed to the manuscript.

## Abbreviations

ERK: extracellular signal-regulated kinase
KTR: kinase translocation reporter
EGF: epidermal growth factor
MEK: mitogen-activated protein kinase
FRET: Förster resonance energy transfer
EKAREV: extracellular signal-regulated kinase activity reporter-Eevee-linker
FLIM: fluorescence lifetime imaging microscopy
