# Supplementary material for: A sensitive ERK fluorescent probe reveals the significance of minimal EGF-induced transcription
Source: Cell Struct Funct. 2024 Dec 18;50(1):15–24. doi: 10.1247/csf.24070 (PMC12706509; doi:10.1247/csf.24070)
Supplement: Supplementary file 1 — Supplementary Figures [file csf_50_24070_1.zip › 50_24070_Fig_S1_S2..docx]

Supplementary Materials for

**A sensitive ERK fluorescent probe reveals the significance of minimal EGF-induced transcription**

Zhang Weisheng *et al.*

*Corresponding author. Email: [toru.hiratsuka@oici.jp](mailto:toru.hiratsuka@oici.jp)

**This file includes:**

Supplementary figures: Figs. S1 to S2


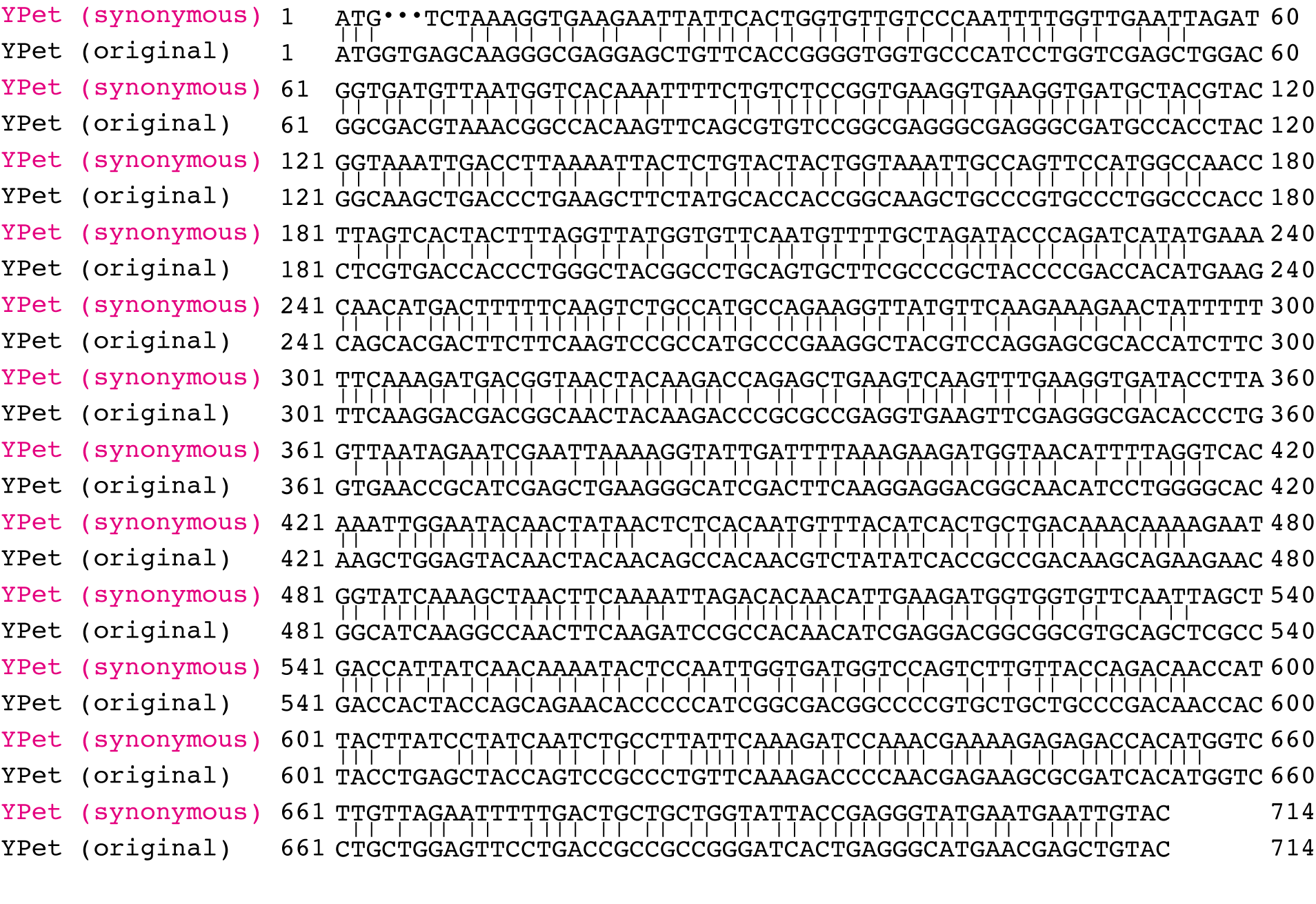


Fig. S1 Comparison of the original YPet sequence in EKAREN5 and a synonymous codon variant YPet sequence in EAKREN5-gl.


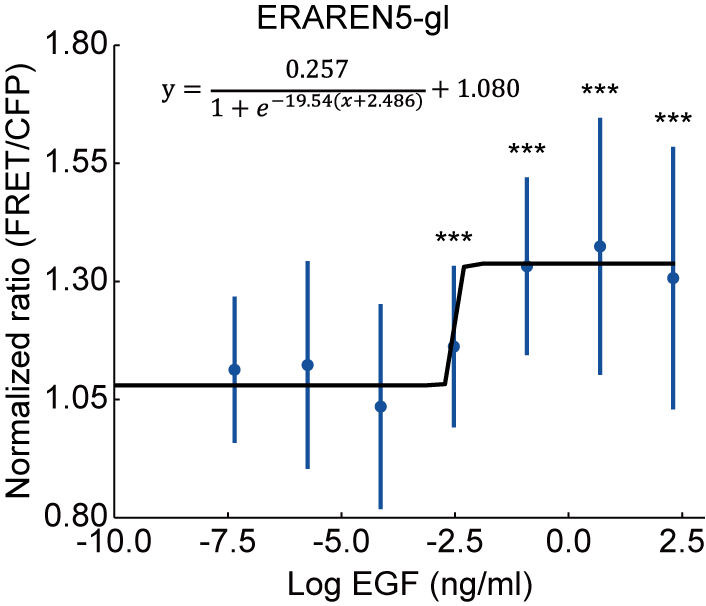


Fig. S2 Dose-dependent response of EKAREN5-gl to EGF-indued ERK activations. The peak FRET ratios (FRET/CFP) in HeLa cells expressing EKAREN5-gl after EGF treatment are shown. Cells were treated with the indicated dose of EGF at 10 min. The values are normalized by the average FRET ratio before EGF addition. Data are shown by mean ± SD. Black line shows a sigmoid curve fitting (fitting function is shown above).
